# Supplementary material for: A novel causality-based method for identifying drivers of breast cancer progression
Source: Bioinformatics. 2026 Sep 11;42(9):btag661. doi: 10.1093/bioinformatics/btag661 (PMC13587825; doi:10.1093/bioinformatics/btag661)
Supplement: Supplementary file 1 [file btag661_supplementary_data.zip › Supplementary Materials.pdf]

Bioinformatics, , pp. 1–10

doi:
Original Paper

ORIGINAL PAPER

A Novel Causality-Based Method for Identifying Drivers of Breast Cancer Progression

Lai Shen[1,†], Yinghao Zhang[1,†], Xiaoyan Zhou[1,†], Jiuyong Li[2], Lin Liu[2], Wen Zhang[1,4], Hong-Yu Zhang[1,4], Xiaomei Li[5,*], Debo Cheng[3,*] and Zaiwen Feng[1,4,*]

[1] College of Informatics, Huazhong Agricultural University, 430070, Wuhan, China , [2] College of Engineering and Information Technology, Adelaide University, 5005, South Australia, Australia , [3] School of Computer Science and Technology, Hainan University, 570228, Haikou, China , [4] Hubei Key Laboratory of Agricultural Bioinformatics, Huazhong Agricultural University, 430070, Wuhan, China and [5] Business Banking, Commonwealth Bank, 2000, NSW, Australia

[*] Corresponding authors: xiaomei.hfut@gmail.com (Xiaomei Li), chengd@hainanu.edu.cn (Debo Cheng), Zaiwen.Feng@mail.hzau.edu.cn (Zaiwen Feng)

[†] These authors contributed equally to this work.

Abstract

Motivation: Identifying transcriptomic factors with potential causal effects on breast cancer progression is important for understanding disease mechanisms and prioritizing therapeutic targets. However, causal-effect estimation from high-dimensional gene-expression data remains challenging because of the large number of variables and potential unmeasured confounding.

Results: We propose CICIV, a causal inference framework that integrates PC-simple-based causal feature selection with conditional instrumental variable (CIV) representation learning. PC-simple first reduces the dimensionality of transcriptomic data by identifying candidate parent genes, after which CIV estimates and ranks their absolute causal effects. Applied to TCGA-BRCA, CICIV prioritized 40 breast cancer-related candidate genes and identified signals that were not captured by conventional correlation-based analyses. External validation using the independent METABRIC cohort showed consistent effect directions for 21 of the 40 genes, with four genes overlapping in the Top 10 and nine in the Top 20 rankings. Pathway enrichment and literature-based analyses further supported the biological relevance of the prioritized genes.

Availability and Implementation: The CICIV benchmarking framework and source code are freely available at `https://github.com/Zaiwen/CICIV`.The software version and test data used in this study are archived at Zenodo (DOI: 10.5281/zenodo.22143976).

Contact: xiaomei.hfut@gmail.com; chengd@hainanu.edu.cn; Zaiwen.Feng@mail.hzau.edu.cn

Supplementary Information: Supplementary data are available at *Bioinformatics* online.

Key words: Breast Cancer, Causal Effect Estimation, Genes, Latent Confounders, Transcriptomic Data

Introduction

Breast cancer is the most common cancer in women and the leading cause of cancer-related deaths among women globally, as reported by Global Cancer Statistics 2020 (Cao et al., 2021). With an estimated 2.3 million new cases and 685,000 deaths annually, the incidence of breast cancer has risen in most countries over the past decades due to increased life expectancy and lifestyle changes (Porter, 2009). A vital approach to advancing cancer genomic research is the identification of cancer driver genes (Raphael et al., 2014). Gene expression data, typically measured through techniques such as microarrays or RNA sequencing (RNA-seq), captures the activity levels of genes under different conditions. Understanding the expression of the genes under study is essential for developing effective cancer interventions.

One major challenge in analyzing gene expression data lies in its high dimensionality. The large number of variables (i.e., genes) and their interdependencies often complicate analysis and may degrade the performance of algorithms (Flores et al., 2013). Common dimensionality reduction techniques, such as Principal Component Analysis (PCA), t-SNE, and Lasso regression, are frequently used to tackle this challenge. However, these methods often involve iterative addition or deletion of rows and columns or focus solely on a single data quality metric (Yang et al., 2003). Such approaches risk distorting the data or introducing biases, failing to achieve effective dimensionality reduction while maintaining critical relationships. To overcome these limitations, a new method is needed to reduce the dimensionality of gene transcription data efficiently, without introducing distortions.

© The Author . Published by Oxford University Press. All rights reserved. For permissions, please e-mail: journals.permissions@oup.com

Causal inference, a fundamental goal in scientific research across fields like medicine, economics, and behavioral sciences, often seeks to determine the causal effects of treatments on outcomes (Imbens and Rubin, 2015; Pearl, 2009). A primary challenge in causal inference is addressing confounding factors. While randomized controlled trials (RCTs) are the gold standard for eliminating confounders, they are often impractical in real-world settings due to ethical, logistical, and time constraints (Cartwright, 2010; Imbens and Rubin, 2015).

Most data-driven approaches rely on the nonconfoundedness assumption, which is challenging to satisfy in real-world datasets with latent confounders (Pearl, 2009; Cheng et al., 2024a,b). The estimation of absolute conditional average causal estimates (EATE) from the dataset is an evaluative factor for causal inference, with the magnitude of EATE indicating the magnitude of causal strength present between variables. Instrumental variables (IV) offer a promising method to mitigate confounding bias, but their applicability is limited by strict validity conditions, making it difficult to identify valid IVs in practice (Cheng et al., 2023a,b). This creates a pressing need for alternative methods for identifying valid instrumental variables in the presence of latent confounders. This is used as a basis for estimating EATE to estimate causal effects unbiasedly from confounded data.

In this paper, we propose a novel data-driven algorithm for causal inference, named CICIV (Causal Inference with Conditional Instrumental Variables). The CICIV algorithm utilizes causal discovery for the downscaling process of high level genetic data. First, the CICIV algorithm discovers relevant genes by causally inferring gene expression after preprocessing, specifically by utilizing the first stage of the CICIV algorithm's PC-simple module, which performs a conditional independence test on the data, thus ensuring efficient dimensionality reduction (Nogueira et al., 2022). Next, CICIV leverages disentangled representation learning to identify conditions serving as valid IV and estimates the absolute average treatment effects (EATE) of treatments on outcomes in the presence of latent confounding factors (Cheng et al., 2023a,b).

Our CICIV approach builds on the theoretical insights in the work by Bühlmann et al. (Bühlmann et al., 2010), which demonstrated the utility of conditional independence assumptions in identifying variables with strong correlations to outcome variable. Inspired by these results, we use the PC-simple module to introduce conditional independence tests and construct gene regulatory networks for gene expression data to identify key drivers of causal significance. To estimate average causal effects, CICIV utilises CIV, a loosely conditioned form of IV. CIV reduces the restrictive conditions traditionally imposed on IVs and enhances causal effect estimation by utilizing disentangled representations (Cheng et al., 2023a). These innovations provide a solid foundation for studying the causal impact of gene expression on disease progression.

Specifically, we make the following contributions.

- We introduce a new method that utilizes the first stage PC-simple module of the CICIV algorithm to efficiently reduce the size of high-dimensional genetic data. This method enables the identification of key genes while preserving critical causal relationships.
- We address the challenging task of estimating absolute average treatment effects (EATE) in the presence of latent confounders. By learning to optimize traditional IV instrumental variables using CIV de-entangled representations, our approach provides robust and accurate causal effect estimates.
- Through extensive experiments on breast cancer transcriptome datasets, we have demonstrated that our algorithm is able to discover causal relationships that cannot be identified by traditional methods and identify potential drivers of breast cancer, with results that are consistent with and confirmed by findings from a large body of existing literature.

Related Work

In this section, we review prior research relevant to our proposed CICIV method.

In practice, High-dimensional gene expression data usually require dimensionality reduction before downstream analysis. Previous studies have used methods such as PCA, NMF, deep representation learning, and Transformer-based feature extraction to reduce data dimensions and improve classification or prediction performance (Maćkiewicz and Ratajczak, 1993; Tsuge et al., 2001; Choi et al., 2016; Ray et al., 2021; Han et al., 2024). However, these methods mainly focus on statistical feature extraction and may fail to preserve causal regulatory relationships among genes. The PC-simple algorithm is closely related to our work because it uses conditional independence tests for causal feature selection and can effectively reduce the combinatorial complexity of high-dimensional causal discovery (Bühlmann et al., 2010; Nogueira et al., 2022). Compared with traditional dimensionality reduction methods such as PCA, t-SNE, and Lasso, PC-simple performs supervised causal dimensionality reduction by iteratively removing conditionally independent variables, which helps identify candidate parent genes of the target gene while preserving possible causal associations. In cancer transcriptomic studies, several works have attempted to infer causal relationships from gene expression or proteomic data (Pe'er et al., 2001; Tran et al., 2011), but they often do not fully address confounding bias, especially unmeasured confounders, which may cause correlation to be mistaken for causation. To overcome these limitations, our CICIV framework integrates PC-simple-based causal feature selection with CIV-based causal effect estimation in a unified pipeline. PC-simple first screens key genes from large-scale transcriptomic data, reducing noise and computational burden, while the CIV module uses disentangled representation learning to relax the strict assumptions of traditional instrumental variable methods and model potential latent confounders. Therefore, CICIV can achieve the whole-process analysis from causal structure discovery to causal effect quantification, making it more suitable for identifying potential breast cancer driver genes from observational gene expression data.

Materials and Methods

Datasets

In this study, we used TCGA-BRCA transcriptomic data from The Cancer Genome Atlas (TCGA), focusing on HTSeq-Counts gene expression profiles aligned to the GRCh38 (hg38) reference genome (Pan et al., 2019). Samples with clearly annotated vital status were retained and classified according to the TCGA sample type coding system, including primary tumor (TP) and solid tissue normal (NT) samples. Gene expression data were obtained using the *TCGAbiolinks* package, and the final TCGA cohort included 1,214 samples, consisting of 1,101 TP samples and 113 NT samples.

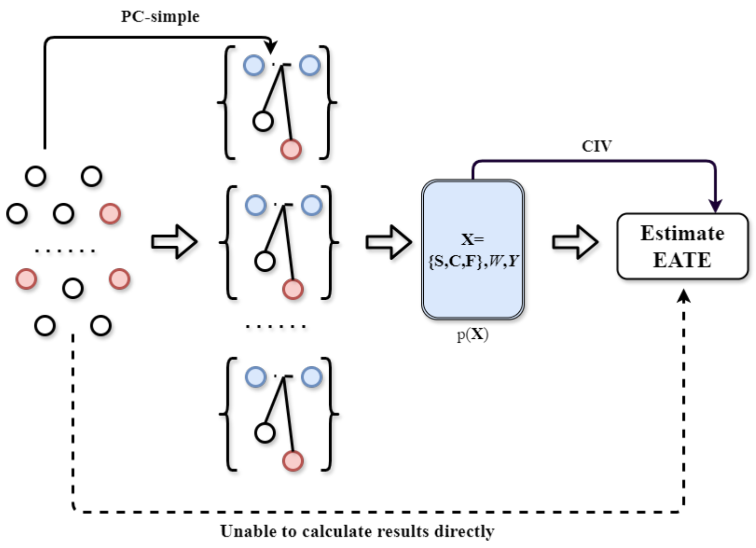

Fig. 1. The architecture of the CICIV model.

For preprocessing, gene expression values were standardized by GC content and gene length to reduce technical bias. Low-expression genes were removed by quantile filtering, and logarithmic transformation was applied to improve distributional normality. After preprocessing, expression profiles of 23,193 genes were retained. We then identified and refined candidate target genes based on unsupervised learning, tumor molecular subtypes, and literature evidence. Ultimately, 40 breast cancer progression-related target genes from the Cancer Gene Census database (`https://cancer.sanger.ac.uk/census`) were selected for subsequent causal inference analysis.

To evaluate the generalization capability of CICIV, we further used the independent METABRIC breast cancer dataset from cBioPortal (`https://www.cbioportal.org/study/summary?id=brca_metabric`). METABRIC contains Illumina microarray-based gene expression profiles from 1,980 breast cancer patients with clinical follow-up information. Expression values of the same 40 target genes were extracted and discretized into low, medium, and high expression levels using quantile-based thresholds. Because TCGA and METABRIC differ in sequencing platforms, sample sources, and population composition, their comparison provides a rigorous setting for assessing the cross-dataset robustness of the CICIV framework.

Methods

Overview

To effectively address high-dimensional datasets where the number of variables exceeds the number of samples, we employed independence tests to identify a small subset of key regulatory factors and construct a causal graph representing potential regulatory relationships among genes. This approach efficiently selects variables relevant to the target gene, provides an intuitive causal structure, and achieves dimensionality reduction.

For directed acyclic graphs (DAGs), in this study, we leverage causal reasoning and verified deep variational autoencoders (DVAE) to extract latent variable information from complex pathways in DAGs. By utilizing the advantages of disentangled representation learning, we estimate absolute causal effects with increased accuracy.

These two components are seamlessly integrated into the proposed framework(Fig. 1), forming a multistage causal reasoning model with the following stages:

- **Identification of the Parents of the Target Gene**: Key genes and their potential causal effects are identified from gene expression data. Using these results, a causal graph is constructed to represent latent gene regulatory relationships.
- **Estimation of Absolute Treatment Effects (EATE) through CIVs**: Latent variable information from complex DAG pathways is extracted iteratively to identify effective CIVs, eliminating confounding bias. This process enables the estimation of the average treatment effect (ATE) and individual treatment effect (ITE), ultimately calculating the absolute average causal effect from data containing potential confounders.

Identify Parents for Target Genes

This process involves reducing the dimensionality of the data and extracting conditionally independent variables as covariates for downstream analysis. To identify the parent genes of a target gene, the input consists of a matrix of gene expression data, where each element represents the expression level of a gene, typically measured as a count or normalized value e.g., TPM (transcripts per million) or FPKM (transcript fragments per kilobase per million mapped reads). The goal is to identify independent variables that have a direct effect on the target gene.

The procedure starts with a complete undirected graph and iteratively removes edges $X - Y$ where $X \amalg Y \mid Z$, for some (potentially empty) conditioning set Z. This process begins with an empty conditioning set and incrementally increases its size until conditional independence $X \amalg Y \mid \{\}, \forall$ other pairs (X, Y), $X \amalg Y \mid \{C\}$.

Conditional independence tests are performed for each variable to determine whether it is independent of the target variable under a given set of conditions. The PC set is updated

using the following statistic:

$$Z = \frac{1}{2} ln(\frac{1+r}{1-r}) \sqrt{n - |S| - 3} \quad (1)$$

where r is the correlation coefficient between X and Y conditioning on a given set S. For continuous variables, r is computed as:

$$r = \frac{Cov(R_X, R_Y)}{\sqrt{Var(R_X) \cdot Var(R_Y)}} \quad (2)$$

The regression model for Y and X are given as

$$Y == S\beta_Y + R_Y \quad (3)$$

$$X = S\beta_X + R_X \quad (4)$$

where R_X and R_Y are the residuals of X and Y, respectively:

$$R_X = X - S\hat{\beta_X} \quad (5)$$

$$R_Y = Y - S\hat{\beta_Y} \quad (6)$$

Identifying immoralities (v-structures) is one of the core challenges of the algorithm. For any paths $X - Z - Y$ in the working graph, the following conditions are used to determine whether a v-structure exists:

1. There is no direct edge between X and Y in the graph, as determined in the previous step.

2. Z is not included in the conditioning set that renders X and Y conditionally independent.

If these conditions are satisfied, $X - Z - Y$ forms an immortality, i.e., a v-structure. The identified v-structures help to orient the edges of the graph and to refine the causal structure. The process of identifying the causal skeleton, including the detection of v-structures, is illustrated in Fig. 2.

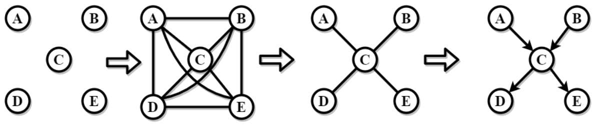

Fig. 2. Identifying the causal skeleton

Next, we leverage the discovered immoralities to orient additional edges in the causal graph. Specifically, any edge $Z - Y$ that is part of the structure $X \to Z - Y$, where there is no edge connecting X and Y can be oriented as $Z \to Y$. This approach systematically refines the directional relationships within the graph. The updated causal structure is depicted in Fig. 3.

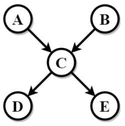

Fig. 3. The true graph

If a variable is found to be independent of the target variable under a given set of conditions, it is removed from the PC set. The stability of the PC set is determined using the following process:

1. If the PC set remains unchanged after an iteration, the algorithm terminates.
2. If changes occur in the PC set, the conditional independence tests continue.

Once the process stabilizes, the final PC set is output to construct a causal graph, representing the relationships between variables. The variables remaining in the PC set serve as the candidate parent variables of the target variable.

The resulting causal graph provides a clear depiction of the cause-and-effect relationships among the variables. The first phase flowchart of the CICIV framework, which outlines this process, is illustrated in Fig. 4.

Assumptions and interpretation of the PC-simple module

The PC-simple procedure relies on standard assumptions of PC-style conditional-independence methods, including the causal Markov condition, faithfulness, and causal sufficiency. Under these assumptions, conditional independence relationships in the observed data can be used to infer candidate graphical relationships among variables. However, because transcriptomic data are observational and may contain unmeasured biological and clinical confounders, such as tumor subtype composition, tumor purity, immune infiltration, and microenvironmental variation, the output of PC-simple should not be interpreted as a fully validated causal network.

In the CICIV framework, PC-simple is used primarily as a causal feature-selection and dimensionality-reduction step. Specifically, it identifies candidate parent sets of target genes and provides a reduced set of covariates for downstream causal-effect estimation. Therefore, the PC-simple results are interpreted as candidate regulatory relationships under the assumptions of the model, rather than definitive evidence of direct biological causality.

Estimate Causal Effects by Learning Conditional Instrumental Variable Representation

In the second stage of the CICIV framework, we identify an effective conditional instrumental variable (CIV) to mitigate the influence of confounding bias on causal inference and estimate causal effects. The expression values of "parent" genes and the outcome variable, as depicted in the causal diagram, are utilized as covariates for the algorithm's input. The algorithm outputs the absolute causal effect of these covariates on the target gene.

We show that relaxed IVs can satisfy the conditions for instrumental variables, rendering them valid for use. By employing a machine learning-based approach called Variational Autoencoders (VAEs), we propose learning CIV representations and their conditional sets using disentangled representation learning techniques. Specifically, CIV representations $\mathbf{S}$ and its conditional set $\{\mathbf{C}, \mathbf{F}\}$, we use $\mathbf{S}$ as an effective IV conditional on $\{\mathbf{C}, \mathbf{F}\}$ to estimate the conditional average causal effect of W on Y in data with potential confounders. The process of modeling is expressed as follows:

$$p(\mathbf{X}, W, Y \mid \mathbf{Z}) = p(W \mid \mathbf{Z}_W)\, p(Y \mid \mathbf{Z}_Y, W)\, p(\mathbf{X} \mid \mathbf{Z}_W, \mathbf{Z}_Y) \quad (7)$$

where $\mathbf{Z}_W$ and $\mathbf{Z}_Y$ represent latent variables related to the treatment variable W and the outcome variable Y, $\mathbf{Z}_Y$

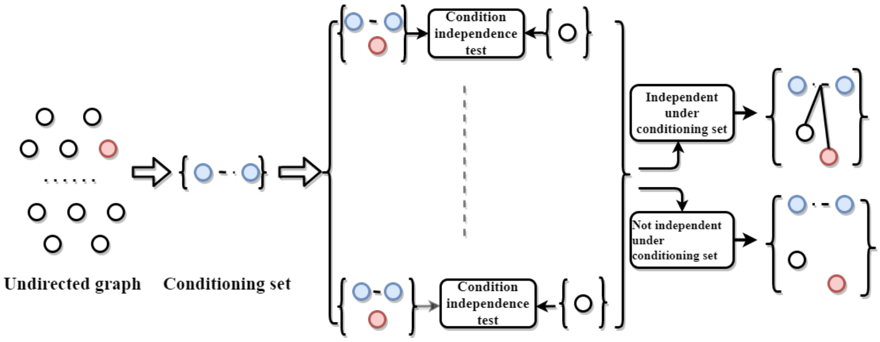

Fig. 4. Flowchart of the first stage of the CICIV framework

represents the confounding factor U. Here, we also need to assume $\mathbf{Z}_W$, $\mathbf{Z}_Y$, $\mathbf{Z}_U$ Independence meets certain conditions:

$$\mathbf{Z}_W \perp \mathbf{Z}_Y \mid \mathbf{Z}_U \quad (8)$$

$$\mathbf{Z}_U \perp \{\mathbf{S}, \mathbf{C}, \mathbf{F}\} \quad (9)$$

The variational posterior distribution $q(\mathbf{Z}_W, \mathbf{Z}_Y, \mathbf{Z}_U \mid \mathbf{X}, W, Y)$ is used to approximate the true posterior distribution $q(\mathbf{Z}_W, \mathbf{Z}_Y, \mathbf{Z}_U \mid \mathbf{X}, W, Y)$. The goal of the variational autoencoder is to maximize the lower bound of evidence (ELBO), and the formula is as follows:

$$\begin{aligned} \text{ELBO} = \; & E_{q(\mathbf{Z}_W, \mathbf{Z}_Y, \mathbf{Z}_U \mid \mathbf{X}, W, Y)} [\log p(W, Y \mid \mathbf{Z}_W, \mathbf{Z}_Y) \\ & + \log p(\mathbf{X} \mid \mathbf{Z}_W, \mathbf{Z}_Y)] - \\ & D_{KL} \left(q(\mathbf{Z}_W, \mathbf{Z}_Y, \mathbf{Z}_U \mid \mathbf{X}, W, Y) \parallel p(\mathbf{Z}_W, \mathbf{Z}_Y, \mathbf{Z}_U) \right) \end{aligned} \quad (10)$$

Once we learn the posterior distribution of the latent variable $q(\mathbf{Z}_W, \mathbf{Z}_Y, \mathbf{Z}_U \mid \mathbf{X}, W, Y)$, we can estimate the conditional average causal effect (CATE),

$$\begin{aligned} CATE(x) &= E[Y(1) - Y(0) \mid \mathbf{X} = x] \\ &= E[Y \mid W = 1, \mathbf{X} = x] - E[Y \mid W = 0, \mathbf{X} = x] \end{aligned} \quad (11)$$

In this work, we take the absolute value of CATE as,

$$\begin{aligned} EATE &= |CATE(x)| \\ &= |E[Y \mid W = 1, \mathbf{X} = x] - E[Y \mid W = 0, \mathbf{X} = x]| \end{aligned} \quad (12)$$

With the CIV model, this expectation can be expressed as,

$$E[Y \mid W = w, \mathbf{X} = x] = E_{q(\mathbf{Z}_Y \mid \mathbf{X})} [E[Y \mid \mathbf{Z}_Y, W = w]] \quad (13)$$

Here, $q(\mathbf{Z}_Y \mid \mathbf{X})$ is the latent variable $\mathbf{Z}_Y$ of the posterior distribution, obtained by variational inference.

We also controlled for confounding factors. We explicitly modeled the confounders $\mathbf{Z}_U$ on the CIV basis to ensure that these unobserved confounders can be effectively controlled when estimating the causal effect. Specifically, conditional independence assumed that $\mathbf{Z}_W \perp \mathbf{Z}_Y \mid \mathbf{Z}_U$ ensures that the effects of confounders are correctly separated when calculating causal effects. Therefore, we calculated the causal effect,

$$\begin{aligned} CATE(x) \approx & E_{q(\mathbf{Z}_Y \mid \mathbf{X})} [f(W = 1, \mathbf{Z}_Y)] - \\ & E_{q(\mathbf{Z}_Y \mid \mathbf{X})} [f(W = 0, \mathbf{Z}_Y)] \end{aligned} \quad (14)$$

We introduced some important notations used in this paper. Let $W \in \{0, 1\}$ denote a binary treatment variable, where $W = 1$ means receiving treatment and $W = 0$ means not receiving treatment, for the treatment and control samples, respectively. Y represents the outcome variable of interest and U represents a set of unobserved confounders. We assume that the observed covariates are learnt through three representations, $\mathbf{S}$, $\mathbf{C}$ and $\mathbf{F}$, where $\mathbf{S}$ affects both treatments W and $\mathbf{C}$, $\mathbf{C}$ represents the confounders that affect both W and the outcome Y, and $\mathbf{F}$ represents the risk factors that affect both $\mathbf{C}$ and Y. The set $\mathbf{X}$ is represented by the three sets $\mathbf{S}$, $\mathbf{C}$, $\mathbf{F}$. where $f(W, \mathbf{Z}_Y)$ is the mapping from $\mathbf{Z}_Y$ to Y by neural network or other function approximation methods, the "≈" in the equation is approximated as "=" is desirable.

To ensure that the treatment W can be recovered from the latent representations $\mathbf{S}$ and $\mathbf{C}$ and the outcome Y from the latent representations $\mathbf{C}$ and $\mathbf{F}$, two auxiliary predictors were added and the objective function of CIV can be formalised as follows:

$$\begin{aligned} L_{CIV} = & -L_{ELBO}(\mathbf{X}, W, Y) + \alpha E_q[\log_q(W \mid \mathbf{S}, \mathbf{C})] \\ & + \beta E_q[\log_q(Y \mid W, \mathbf{C}, \mathbf{F})] \end{aligned} \quad (15)$$

The endogenous relationship between the variables leads to a relationship, with arrows indicating that the variables are causally related to each other, see Supplementary Figure S1.

The second phase flow chart of the CICIV framework is shown in Fig. 5. Then mapped to the latent variable space using an encoder to map the input data ($\mathbf{X}$, W, Y) into the latent variable space, which in turn performs a latent representation of the latent variables and distinguishes between the different latent variables, which correspond to the different parts of $\mathbf{S}$, $\mathbf{C}$, and $\mathbf{F}$, by means of a disentanglement technique. The yellow boxes represent the specific divisions that determine $\mathbf{S}$, $\mathbf{C}$, $\mathbf{F}$. In the generative model, we adopt a Monte Carlo (MC) sampling strategy to sample the distribution $\mathbf{C}$ through a conditional variational autoencoder (VAE) network, so that the latent representation of $\mathbf{C}$ can be generated from the distribution $\mathbf{X}$. The arrows represent the process of mapping and de-entanglement. It shows how CICIV reconstructs the treatment variable W, the outcome variable Y, and the covariate $\mathbf{X}$ through the conditional distribution and calculates the lower bound of the evidence of variational autoencoder (ELBO) (Chakravarthy, 2022), thus continuously optimizing the model and obtaining the value of EATE.

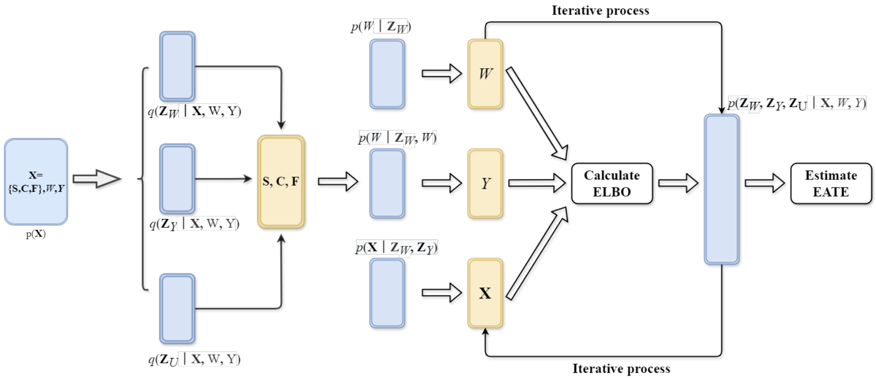

Fig. 5. Flowchart of the second stage of the CICIV framework

CausalEstimand and Interpretation under Cross-sectional Transcriptomic Data

Because TCGA-BRCA tumor/normal transcriptomes were measured after cancer diagnosis, the estimated effects in this study should not be interpreted as definitive evidence that gene-expression alterations initiate breast cancer. Instead, CICIV provides model-based estimates of the association between post-diagnosis gene-expression states and breast cancer/progression-related outcomes under explicit causal assumptions. We acknowledge that gene-expression differences may partly reflect downstream consequences of tumor biology, tumor microenvironment, molecular subtype composition, and other post-onset processes. Therefore, the genes identified by CICIV are interpreted as candidate transcriptomic regulators or causal-effect candidates that require further validation using longitudinal cohorts, perturbation experiments, or functional assays.

Experiments

We continuously pooled data on genes associated with breast cancer disease through a series of experiments to obtain a small data frame containing the target gene. In this experiment, the target variable is usually the state of breast cancer (e.g. whether or not one has breast cancer). The target variables mean the status of breast cancer (0 means not diseased, 1 means diseased); Treatment variables are variables that may have an effect on the target variable, which in this study represents the level of gene expression. We have 40 target genes, and the expression level of each gene can be considered as a treatment variable; Covariates are variables that may affect both the target and treatment variables, and they are used to adjust the analysis to eliminate confounding effects. Common covariates include demographic information (e.g. age, sex), clinical characteristics (e.g., tumor size, stage) (Agarwal et al., 2010), and in this study, other variables that may affect breast cancer status and gene expression levels (e.g., genes in this study).

We obtained 23,192 data sets on breast cancer gene expression. The experimental ideas were divided into two parts. We first conducted experiments to estimate causal effects at the gene expression level, and then conducted comparative experiments on other estimators. In the first part, through a series of analysis of the data, including mean test (CM), correlation analysis (CA), and difference analysis (DEG), we obtained samples of the conditional independence test to estimate the absolute causal effect (EATE) and provide consistent estimates of our method with the results found in previous literature. In the second part, we compared our algorithm with the current advanced estimators.

To reduce the risk that the identified signals simply reflected tumor-versus-normal differences after cancer onset, we further performed a tumor-only progression analysis using clinical progression-related outcomes. Specifically, primary tumor samples were analyzed separately, and progression-related phenotypes, including pathological stage, lymph-node involvement, distant metastasis, and survival outcome, were used as downstream outcomes. This analysis aimed to evaluate whether CICIV-prioritized genes were associated with disease progression among breast cancer patients rather than merely distinguishing tumor tissues from normal tissues.

Although CICIV explicitly models latent confounding, residual confounding from tumor purity, immune infiltration, and subtype composition cannot be fully excluded.

Causal effects on gene expression levels

In this part, we reduced the dimension of the data and estimate the absolute causal effect.

The experiment took Compare Means (CM) to get the p-value of each gene, controlling the p-value to be less than 0.05, and finally we got 18614 genes, we focused on the ordering of the target genes, and it is worth noting that there are only 5 genes outside the value of p-value criterion. They were in descending order of significance, as shown in Supplementary Table S1.

We performed a correlation analysis (CA) of the relationship between genes and breast cancer based on logistic regression modeling to analyze the relationship between gene expression levels and the target variable. OR (Odds ratio) is a metric commonly used in statistical analyses to measure the relative likelihood that an event will occur between two groups. In particular in medicine and biology, OR indicators are commonly used to assess the association between a factor (e.g., gene expression level) and a disease state. In this experiment, controlling for $OR > 1$ and $p - value < 0.05$, and also focusing on the sequencing of target genes, we found that 17 of the 40 target genes showed significant expression by correlation analysis. The rank of their correlation strengths was shown in Supplementary Table S2.

For significant genes in the above mean test and the correlation test, containing 18614, we performed a differential expression analysis of genes (DEG) (Costa-Silva et al., 2017), which is applicable to RNA-Seq data, to identify differentially expressed genes by comparing gene expression levels under

different experimental conditions using the DESeq2 method. To detect significantly differentially expressed genes according to a preset threshold, we controlled for the log2 fold change (Love et al., 2014) of risk factors contributing to breast cancer between the two groups, $|log2FoldChange| > 0.5$ and $adjp < 0.05$, which is considered to be a change in gene expression of about 1.41 fold between the experimental group and the control group in this condition, and the magnitude of this change is already more than 1.3 fold, which is usually considered to have a biological significance. We focused on the sequencing of the target genes, and 18 of the 40 target genes were found to be significantly different by correlation analysis.

The experimental results showed that a positive ATE value indicates that high gene expression was positively correlated with the state of disease and may promote the disease, while a negative ATE value indicated that high gene expression was negatively correlated with the state of the disease and may inhibit the disease. We showed the positive and negative ATE values of the target gene in Supplementary Table S3. Taking the absolute value to get EATE, the larger the EATE value, the stronger the causal relationship. The results showed that the effects of some of these genes have been verified in the literature and that the remaining genes might be good candidate genes for the future treatment of breast cancer. The results of the statistical synthesis were shown in Supplementary Figure S2.

We took the top 20 genes (top 20) based on the absolute causal effect ranking values obtained and compared them with the comparing means (CM), correlation analysis (CA), and differential expression genes (DEG), respectively. The analysis showed that among the top 20 genes, 19 of the top 35 genes in the mean difference comparison ranking were in the known causal relationship ranking; 11 of the top 17 genes in the correlation analysis were in the known causal relationship ranking; and 13 of the top 18 genes in the differential expression analysis were in the known causal relationship ranking. The proportions were 54.3%, 64.7%, 72.2%, respectively, all higher than 50%.

We verified the results of conditional average causal effect by means of a literature search, which was a method often cited by previous scholars in causal studies. The study results showed that 7 genes out of 17 negative ATE results had corresponding literature support, and 15 genes out of 23 positive ATE results had corresponding literature support. The results were summarized in Supplementary Table S4.

External validation of breast cancer causal genes

We externally validated the 40 breast cancer causal genes identified from TCGA-BRCA using the independent METABRIC dataset. For each target gene, the PC-simple algorithm was applied at a significance level of (alpha=0.01) to identify its PC set, which was then used as adjustment variables for estimating the average treatment effect (ATE) on survival outcomes. The METABRIC results were compared with the original TCGA findings in terms of causal-effect direction, numerical consistency, and ranking overlap.

In METABRIC, PC discovery was successfully performed for all 40 target genes, with an average of 2.9 PC members per gene, indicating moderate causal-network connectivity. Except for six genes (EP300, SALL4, NOTCH1, ZMYM3, CDKN1B, and ARID1B), no target gene identified the SURVIVAL variable as a PC member. Network analysis further identified FOXA1, GATA3, and ESR1 as central hub genes, forming a biologically plausible transcriptional regulatory module associated with hormone receptor-positive breast cancer.The results were summarized in Table 1. Survival outcomes typically represent downstream consequences of gene expression changes rather than direct causal neighbors, consistent with our expectations.

Table 1. Statistical Results of PC Causal on METABRIC

Statistical Indicators	numerical value
Verify the total number of genes	40/40 (100% coverage)
Average number of PCs	2.9 per gene
No PC gene count	6
SURVIVAL as the genetic number for PCs	0

Overall, 21 of the 40 genes (52.5%) showed consistent causal-effect directions across TCGA and METABRIC. In addition, 4 genes overlapped in the Top 10 rankings and 9 genes overlapped in the Top 20 rankings between the two datasets. Representative positive-effect genes included AKT1, ESR1, and SALL4, whereas FBLN2, TBX3, PPM1D, TP53, and FLNA showed consistent negative effects. These findings provide preliminary evidence for the cross-dataset robustness of the CICIV framework.

The differences between TCGA and METABRIC may be attributed to both technical and cohort-level heterogeneity. Technically, TCGA uses RNA-seq, whereas METABRIC relies on the Illumina microarray platform, leading to systematic differences in expression quantification and potential platform-specific biases even after normalization. At the cohort level, the two datasets differ in sample sources and population composition, which may further affect the relationship between gene expression and disease outcomes.

GO Enrichment Analysis and Pathway Enrichment Analysis

To further validate the 40 breast cancer driver genes identified by the CICIV framework, we performed pathway enrichment analysis using Enrichr with WikiPathways data (adjusted p-value < 0.05). The dot plot (Supplementary Figure S3) displays the top 20 significantly enriched pathways, including *Breast Cancer Pathway (WP4262)*, *Integrated Cancer Pathway (WP1971)*, *DNA Damage Response Only ATM Dependent (WP710)*, and *ErbB Signaling (WP673)*, all of which are closely related to breast cancer progression. These pathways exhibit high statistical significance and combined enrichment scores, indicating that the CICIV-identified driver genes are highly relevant to breast cancer biology.

The pathway–gene network plot (Supplementary Figure S4) further illustrates the relationships between driver genes and enriched pathways. Core hub genes, including **TP53, PIK3CA, AKT1, CCND1, ERBB2, ESR1, RB1, BRCA2, CDKN1B, and NOTCH1**, appear repeatedly across multiple pathways, indicating central roles in the regulatory network. Functional annotation allows classification of the enriched pathways and core genes into major molecular mechanisms (Table 2)

Comparison experiments at all estimators

We evaluated the proportion of strongly causal genes identified by different estimators, and the comparison results are summarized in Supplementary Table S5. CICIV consistently achieved the best EATE estimation performance, especially

Table 2. Functional Classification of Enriched Pathways and Core Driver Genes

Functional Category	Representative Pathways	Core Driver Genes
Cell Cycle Regulation	Cell Cycle; DNA Damage Response Only ATM Dependent	TP53, CCND1, CDKN1B
PI3K/AKT/ERBB Signaling	ErbB Signaling; Integrated Cancer Pathway	PIK3CA, AKT1, ERBB2
Transcriptional Regulation / EMT	Androgen Receptor Network; Apoptosis	ARID1A, ARID1B, TP53, FOXA1
DNA Damage Repair	DNA Damage Response Only ATM Dependent	BRCA2, TP53
Hormone Receptor Signaling	Breast Cancer Pathway; ESR1 Signaling	ESR1, PIK3CA

Note: The table summarizes the major functional categories, representative enriched pathways, and corresponding core driver genes identified in the pathway enrichment analysis.

Table 3. Proportion of causality and correlation for each estimator

method	Correlation	Causal relation
ElasticNet	0.67	0.33
R-Lasso	0.60	0.40
Logistic Regression	0.75	0.25
PointBiseralr	0.50	0.50
RandomForest	0.67	0.33
RFSHAP	0.67	0.33
Ridge	0.67	0.33
CICIV	0.33	**0.67**

Note: The table shows the proportion of causality and correlation for each estimator. The best performance is highlighted.

among top-ranked genes, demonstrating its effectiveness in causal effect estimation.

The results are visually shown in Supplementary Figure S5. Genes were grouped according to their EATE rankings into the Top 10, Top 20, and Top 30 sets, and the proportion of potential causal genes identified by each estimator was compared. Across all groups, CICIV consistently outperformed the other estimators, further supporting its advantage in identifying potential causal genes.We evaluated the performance of various estimators in identifying correlations and causations, with the results presented in Table 3.

We further evaluated the ratio of causality to correlation for each estimator, as illustrated in Supplementary Figure S6. After excluding genes unrelated to disease status, the remaining results were classified into 'correlation" and 'causal relationship" categories. CICIV showed a stronger ability to identify causal relationships rather than simple correlations.

Compared with ElasticNet, R-Lasso, RFSHRP, Logistic Regression, Ridge, and PointBiseralr, CICIV performed better in detecting causal effects. Even after genes with significant causal relationships were removed, several traditional estimators still ranked target genes highly based on correlation. These results indicate that CICIV can uncover causal signals overlooked by correlation-based methods while reducing the interference of simple associations.

Table 4. Comparison of causal inference methods on TCGA

method	Top 10	Top 20	Top 30
2SLS	0.26	0.34	0.60
PSM	0.46	0.55	0.32
DeepIV	0.20	0.44	0.43
NOTEARS	0.32	0.50	0.62
CICIV	0.30	**0.70**	**0.80**

Note: The proportion of experimental results that found potential causal relationships. The best performance is highlighted.

Our method outperforms other causal inference methods

To fully verify the superiority of the CICIV framework in causal effect estimation of breast cancer genes, we selected classic and cutting-edge causal inference algorithms suitable for observational high-dimensional data for comparison, and excluded algorithms with inapplicable basic assumptions (e.g., RCT-based ATE estimation). The selected comparison algorithms are as follows:

- **2SLS (Two-Stage Least Squares)**: A classic traditional instrumental variable method, used as the basic control for IV-based causal inference.
- **PSM (Propensity Score Matching)**: A classic confounding control method for observational data, which balances the baseline characteristics of the sample by matching propensity scores.
- **DeepIV**: A deep learning-based instrumental variable method that solves the problem of nonlinear confounding in high-dimensional data.
- **NOTEARS**: A gradient-based causal graph learning algorithm with high adaptability to high-dimensional data, which can realize simultaneous causal structure discovery and effect estimation.

The results are presented in Table 4. they show that CICIV achieved the most concentrated distribution of EATE estimation bias, with the lowest median and quartile values, suggesting that it provided the most stable and accurate causal effect estimates. In contrast, the estimation biases of 2SLS and PSM were more widely distributed and included several extreme values. This may be attributed to the strict instrumental variable assumptions required by 2SLS and the inability of PSM to account for unmeasured confounders in transcriptomic data, both of which can lead to larger errors in causal effect estimation. DeepIV reduced the estimation bias to some extent by incorporating deep learning, but its lack of pre-screening for core causal genes made it susceptible to interference from noise genes, resulting in higher bias than CICIV. Although NOTEARS showed certain advantages in computational efficiency, its limited ability to control unmeasured confounders weakened the accuracy of causal effect quantification compared with CICIV.

Conclusion

To advance the study of gene expression and breast cancer at the transcriptomic level, we proposed the CICIV model, addressing key challenges such as handling high-dimensional variables and eliminating confounding bias effects. The core of this framework lies in identifying key regulatory factors through conditional independence testing and mitigating confounding biases, enabling the accurate estimation of

absolute causal effects. To the best of our knowledge, CICIV is the first algorithm to seamlessly integrate dimensionality reduction and causal inference within a unified framework for gene expression analysis, allowing for the discovery of causal relationships beyond mere correlations. Our extensive comparisons with existing algorithms and analyses of relevant literature demonstrate that CICIV consistently outperforms alternative approaches.

The research findings highlight that the CICIV framework significantly enhances the detection of causal associations among disease-related genes. This innovative methodology not only proves to be an effective tool for estimating causal effects across various applications but also serves as a critical advancement for improving our understanding of disease mechanisms, prevention, and treatment strategies.

In conclusion, CICIV provides a causal-inference-based prioritization framework for identifying candidate transcriptomic regulators associated with breast cancer progression. Given the cross-sectional nature of TCGA-BRCA tumor/normal transcriptomes, the identified genes should be interpreted as candidates supported by model-based causal-effect estimates rather than definitive causal drivers of cancer initiation. The consistency observed in the independent METABRIC cohort and pathway enrichment analyses provides supportive evidence for their biological relevance, but further longitudinal and perturbation-based validation is required to establish direct causality.

Future Research

The 40 breast cancer causal genes identified by the CICIV framework, especially the 21 genes with consistent causal effect directions in two independent datasets and the Top20 core causal genes, provide high-quality candidate genes for subsequent breast cancer biological functional verification and clinical translational research, and the specific future research directions are as follows:

- **Biological functional verification of candidate genes**: For the core causal genes (e.g.AKT1,ESR1,FOXA1) and potential novel candidate genes(e.g.IRS4, IKZF3) identified in this study, subsequent cell experiments (e.g.gene knockout/overexpression, Transwell, CCK-8) can be used to verify their regulatory effects on breast cancer cell proliferation, apoptosis, invasion and metastasis; animal experiments (e.g.nude mouse subcutaneous transplantation tumor model, tail vein metastasis model) can be used to verify their in vivo regulatory effects on breast cancer tumor growth and metastasis.
- **Exploration of molecular regulatory mechanisms**: For the core causal gene modules and pathways identified by GO and pathway enrichment analysis (e.g.FOXA1-ESR1-GATA3 transcriptional axis, PI3K-Akt signaling pathway), subsequent studies can explore the specific molecular regulatory mechanisms (e.g.protein-protein interaction, transcriptional regulation) between genes, and clarify the key regulatory nodes in the causal network of breast cancer progression.
- **Development of clinical diagnostic and prognostic markers**: Based on the causal effect of the identified genes on breast cancer, subsequent studies can analyze the correlation between the expression level of these genes and the clinical characteristics of breast cancer patients (e.g.tumor stage, lymph node metastasis, distant metastasis, survival time), and screen out genes with high diagnostic efficiency and prognostic value as potential breast cancer molecular markers, so as to provide a basis for personalized diagnosis of breast cancer.

Data and code availability

The results presented here are based, in whole or in part, on data generated by the TCGA Research Network: `http://www.cancer.gov/tcga`. Finally, this work made the source code and required data supporting this research publicly available via `https://github.com/XiaoyanZhou-hub/CICIV`.

The TCGAbiolinks package in R can be used to extract gene expression data from the TCGA database. For causal structure learning and causal inference, the pcalg package in R, requiring R version 3.5.0, is highly effective. This package includes implementations of various causal structure learning algorithms, particularly the PC-simple algorithm. The PC-simple algorithm leverages conditional independence tests to identify relationships between the target variable and other variables based on a specified alpha significance level. This methodology effectively identifies relevant variables that are conditionally independent of others, facilitating the construction of causal diagrams.

For differential expression analysis, the DESeq2 package is available in R and also requires R version 3.5.0.

Additionally, we also provide a Python script that implements DVAE.CIV based on the CIV causal inference framework. By integrating these scripts with the variable selection process of the PC-simple algorithm, causal effects can be estimated using the selected variables as input features, even when working with synthetic data.

Key Points

- We propose a new method, called CICIV, which utilises the PC-simple module to adequately downscale high-dimensional gene data. This approach identifies key genes while preserving key causal relationships.
- We are committed to tackling the challenging task of estimating the Estimate of Absolute Treatment Effect (EATE) in the presence of potential confounding factors. By learning and optimizing conventional instrumental variables (IV) through a CIV decoupling representation, our method delivers robust and precise causal effect estimates.
- Through extensive experiments on breast cancer transcriptomic datasets, we demonstrate that our algorithm uncovers causal relationships unrecognized by conventional methods and identifies potential drivers of breast cancer. Experimental results align with and corroborate findings from a substantial body of existing literature, including twelve studies based on clinical data.

Acknowledgements

This research project was supported in part by the Major Science and Technology Project of Hubei Province under Grant 2024BAA008.

References

M. Agarwal, G. Brahmanday, G. W. Chmielewski, R. J. Welsh, and K. P. Ravikrishnan. Age, tumor size, type of surgery, and gender predict survival in early stage (stage i and ii) non-small cell lung cancer after surgical resection. *Lung Cancer*, 68(3):398–402, 2010.

P. Bühlmann, M. Kalisch, and M. H. Maathuis. Variable selection in high-dimensional linear models: partially faithful distributions and the PC-simple algorithm. *Biometrika*, 97 (2):261–278, 2010.

W. Cao, H.-D. Chen, Y.-W. Yu, N. Li, and W.-Q. Chen. Changing profiles of cancer burden worldwide and in china: a secondary analysis of the global cancer statistics 2020. *Chinese Medical Journal*, 134(7):783–791, 2021.

N. Cartwright. What are randomised controlled trials good for? *Philosophical Studies*, 147(1):59–70, 2010.

H. B. Chakravarthy. Variational auto-encoders (VAEs): A review, 2022. Final paper for Statistics 185, Harvard College.

D. Cheng, Z. Xu, J. Li, L. Liu, T. D. Le, and J. Liu. Learning conditional instrumental variable representation for causal effect estimation. In *Joint European Conference on Machine Learning and Knowledge Discovery in Databases*, pages 525–540. Springer, 2023a.

D. Cheng, Z. Xu, J. Li, L. Liu, J. Liu, and T. D. Le. Causal inference with conditional instruments using deep generative models. In *Proceedings of the AAAI Conference on Artificial Intelligence*, volume 37, pages 7122–7130, 2023b.

D. Cheng, J. Li, L. Liu, J. Liu, and T. D. Le. Data-driven causal effect estimation based on graphical causal modelling: a survey. *ACM Computing Surveys*, 56(5):1–37, 2024a.

D. Cheng, Z. Xu, J. Li, L. Liu, J. Liu, and T. D. Le. Conditional instrumental variable regression with representation learning for causal inference. In *The Twelfth International Conference on Learning Representations*, 2024b.

Y. Choi, C. Y.-I. Chiu, and D. Sontag. Learning low-dimensional representations of medical concepts. *AMIA Summits on Translational Science Proceedings*, 2016:41, 2016.

J. Costa-Silva, D. Domingues, and F. M. Lopes. RNA-Seq differential expression analysis: an extended review and a software tool. *PLoS ONE*, 12(12):e0190152, 2017.

J. L. Flores, I. Inza, P. Larranaga, and B. Calvo. A new measure for gene expression biclustering based on non-parametric correlation. *Computer Methods and Programs in Biomedicine*, 112(3):367–397, 2013.

Y. Han, L. Han, X. Shi, J. Li, X. Huang, X. Hu, C. Chu, and Z. Geng. Novel cnn-based transformer integrating boruta algorithm for production prediction modeling and energy saving of industrial processes. *Expert Systems with Applications*, page 124447, 2024.

G. W. Imbens and D. B. Rubin. *Causal Inference in Statistics, Social, and Biomedical Sciences.* Cambridge University Press, Cambridge, 2015.

M. I. Love, W. Huber, and S. Anders. Moderated estimation of fold change and dispersion for RNA-seq data with DESeq2. *Genome Biology*, 15:1–21, 2014.

A. Maćkiewicz and W. Ratajczak. Principal components analysis (pca). *Computers & Geosciences*, 19(3):303–342, 1993.

A. R. Nogueira, A. Pugnana, S. Ruggieri, D. Pedreschi, and J. Gama. Methods and tools for causal discovery and causal inference. *Wiley Interdisciplinary Reviews: Data Mining and Knowledge Discovery*, 12(2):e1449, 2022.

B. Pan, R. Kusko, W. Xiao, Y. Zheng, Z. Liu, C. Xiao, S. Sakkiah, W. Guo, P. Gong, C. Zhang, et al. Similarities and differences between variants called with human reference genome hg19 or hg38. *BMC Bioinformatics*, 20:17–29, 2019.

J. Pearl. *Causality.* Cambridge University Press, Cambridge, 2009.

D. Pe'er, A. Regev, G. Elidan, and N. Friedman. Inferring subnetworks from perturbed expression profiles. *Bioinformatics*, $17(suppl_1): S215--S224, 2001.$

P. L. Porter. Global trends in breast cancer incidence and mortality. *Salud Publica de Mexico*, 51:S141–S146, 2009.

B. J. Raphael, J. R. Dobson, L. Oesper, and F. Vandin. Identifying driver mutations in sequenced cancer genomes: computational approaches to enable precision medicine. *Genome Medicine*, 6:1–17, 2014.

P. Ray, S. S. Reddy, and T. Banerjee. Various dimension reduction techniques for high dimensional data analysis: a review. *Artificial Intelligence Review*, 54(5):3473–3515, 2021.

L. M. Tran, B. Zhang, Z. Zhang, C. Zhang, T. Xie, J. R. Lamb, H. Dai, E. E. Schadt, and J. Zhu. Inferring causal genomic alterations in breast cancer using gene expression data. *BMC Systems Biology*, 5:1–13, 2011.

S. Tsuge, M. Shishibori, S. Kuroiwa, and K. Kita. Dimensionality reduction using non-negative matrix factorization for information retrieval. In *2001 IEEE International Conference on Systems, Man and Cybernetics. e-Systems and e-Man for Cybernetics in Cyberspace*, volume 2, pages 960–965. IEEE, 2001.

J. Yang, H. Wang, W. Wang, and P. Yu. Enhanced biclustering on expression data. In *Third IEEE Symposium on Bioinformatics and Bioengineering, 2003. Proceedings*, pages 321–327. IEEE, 2003.
